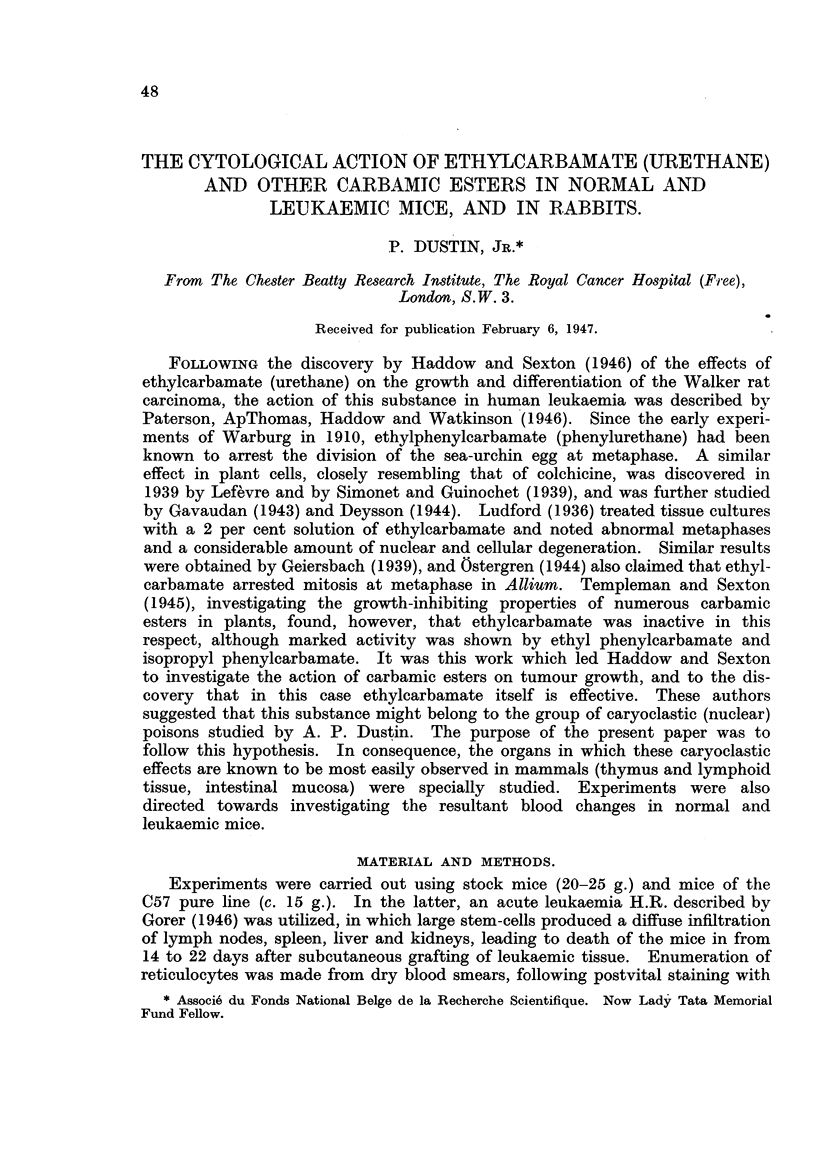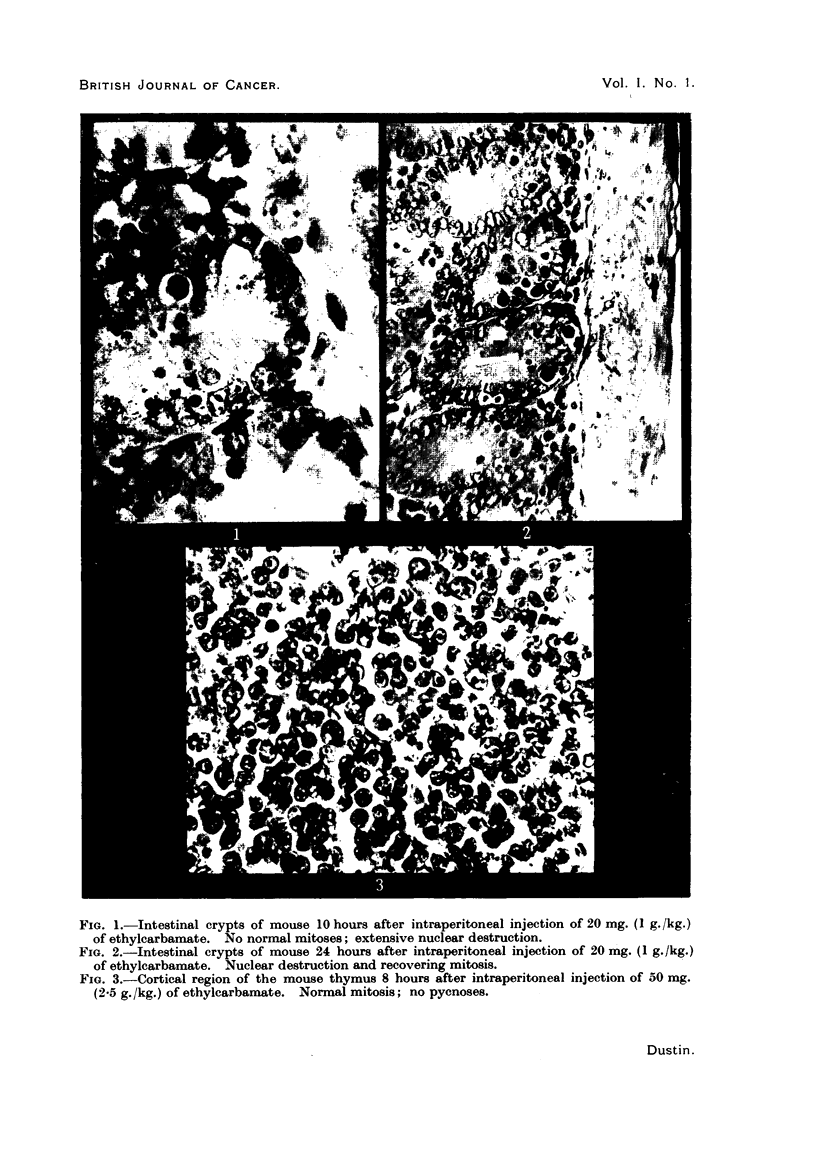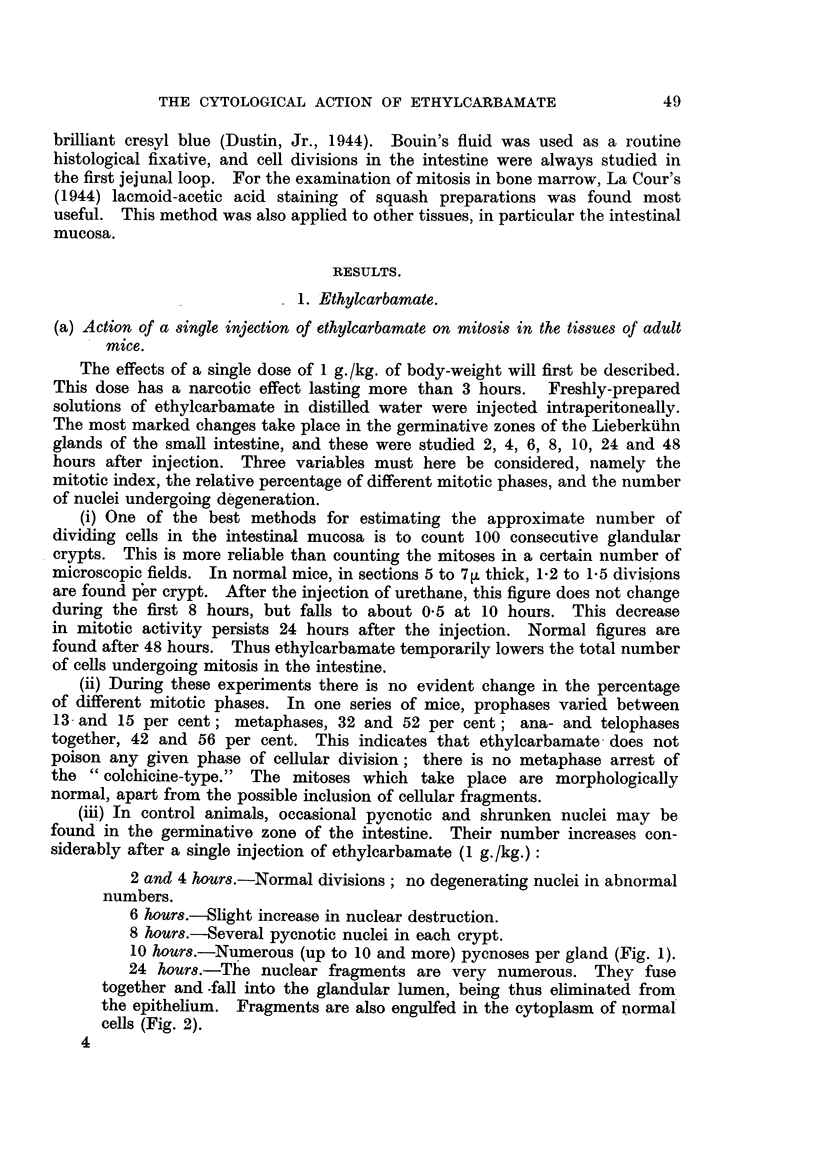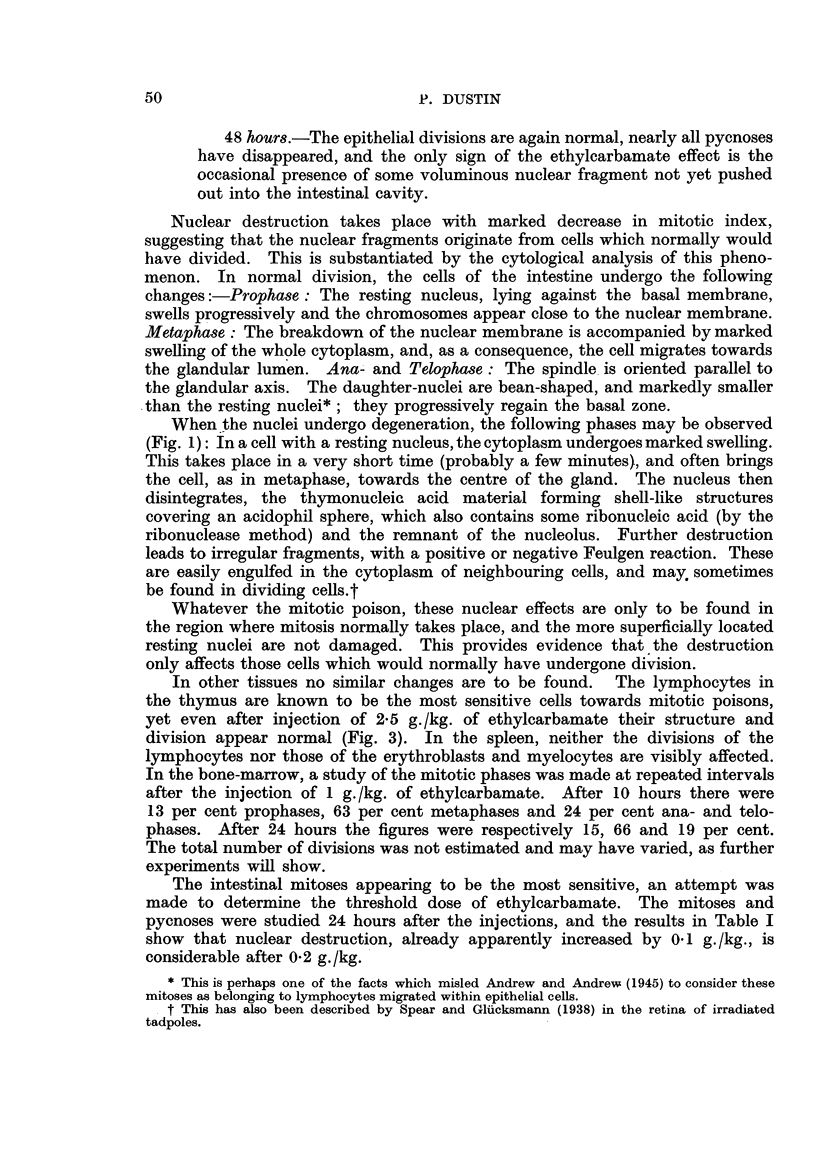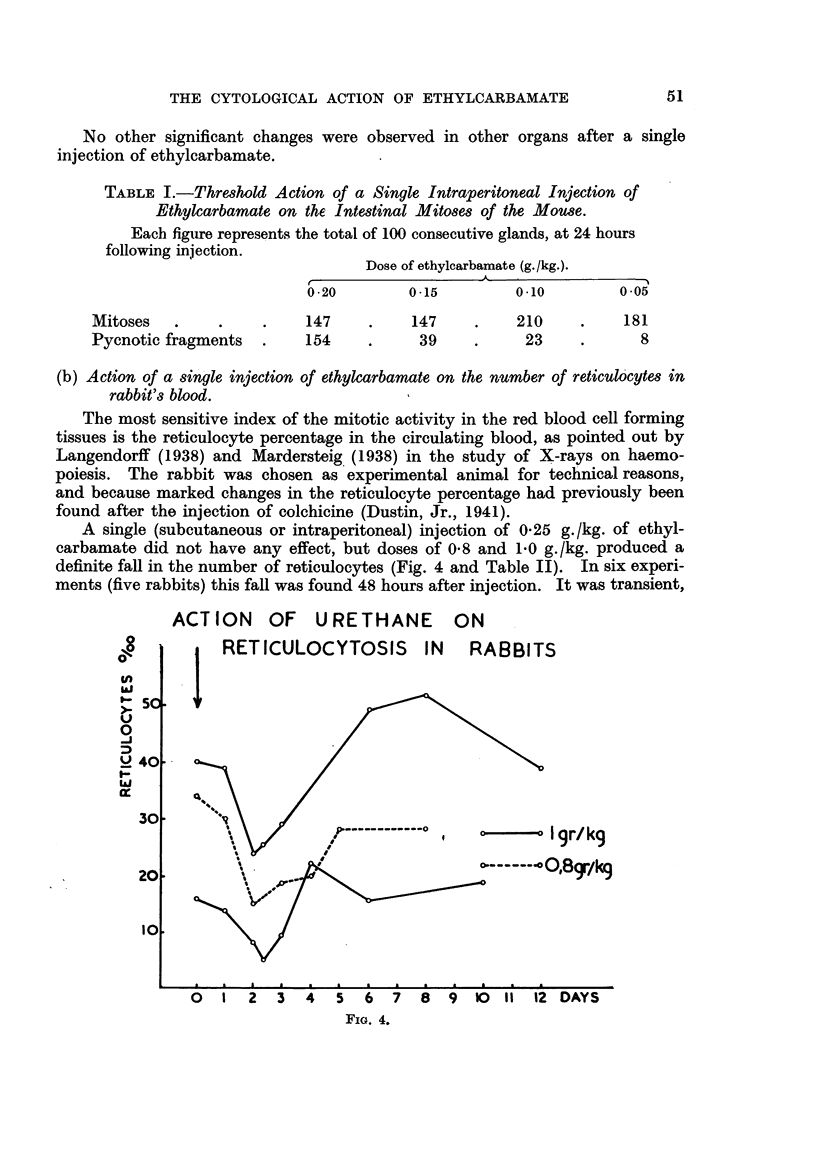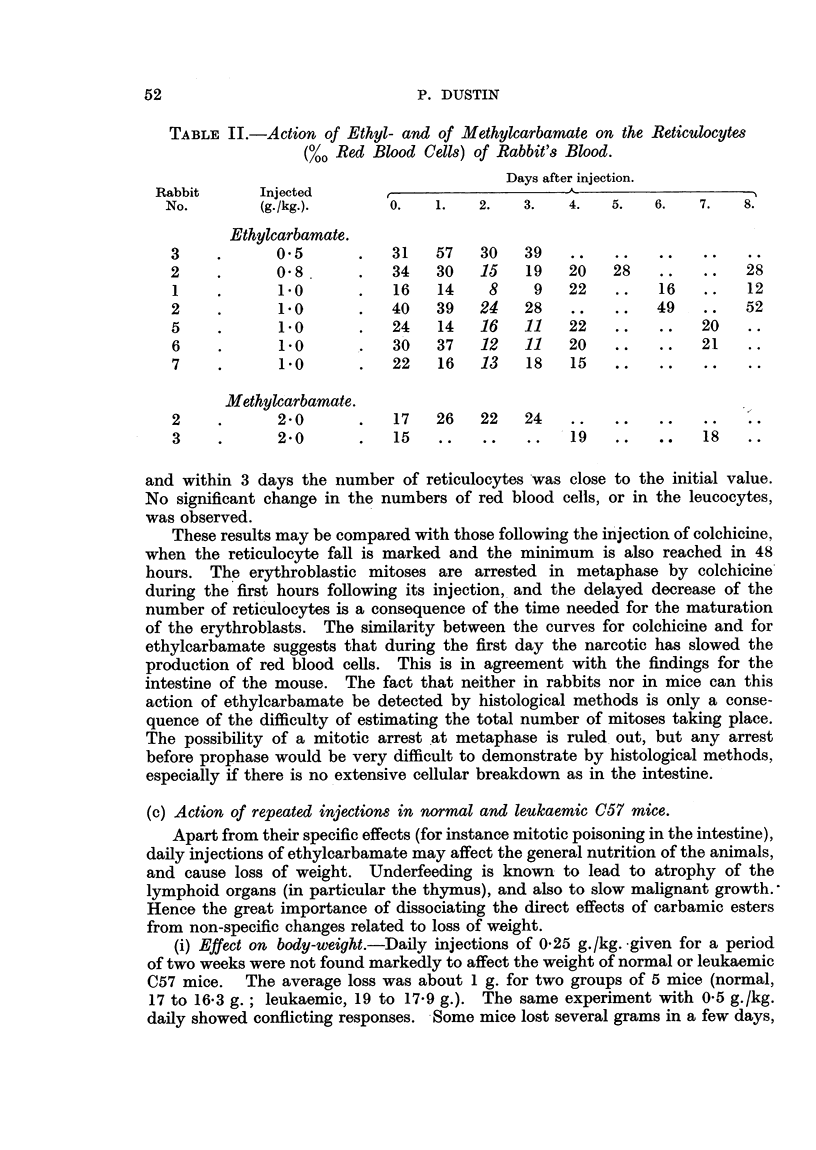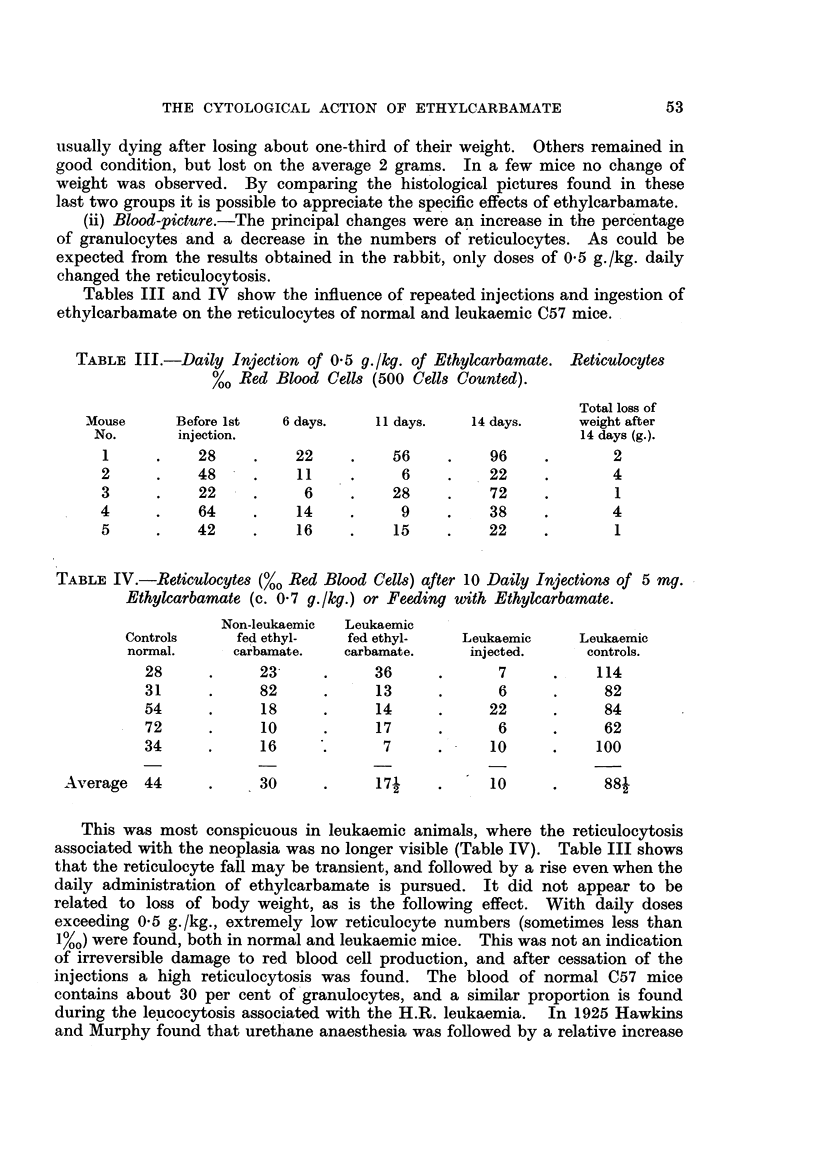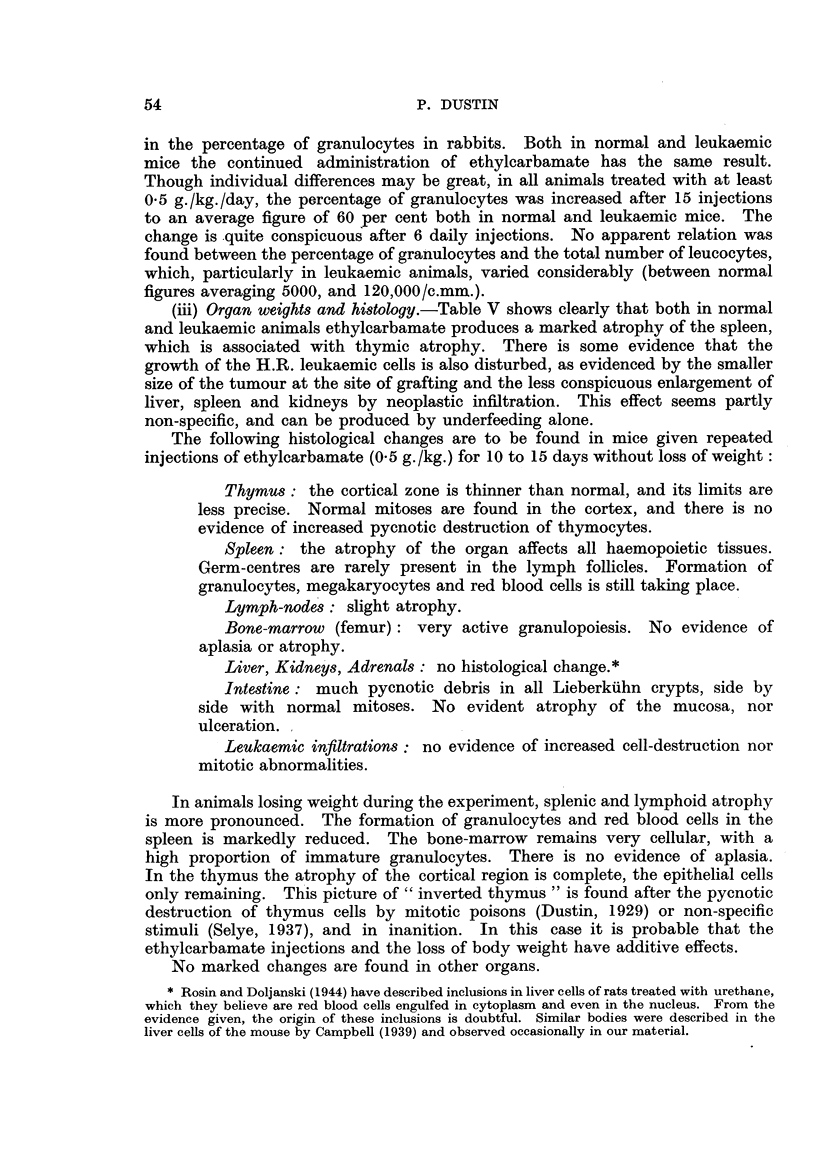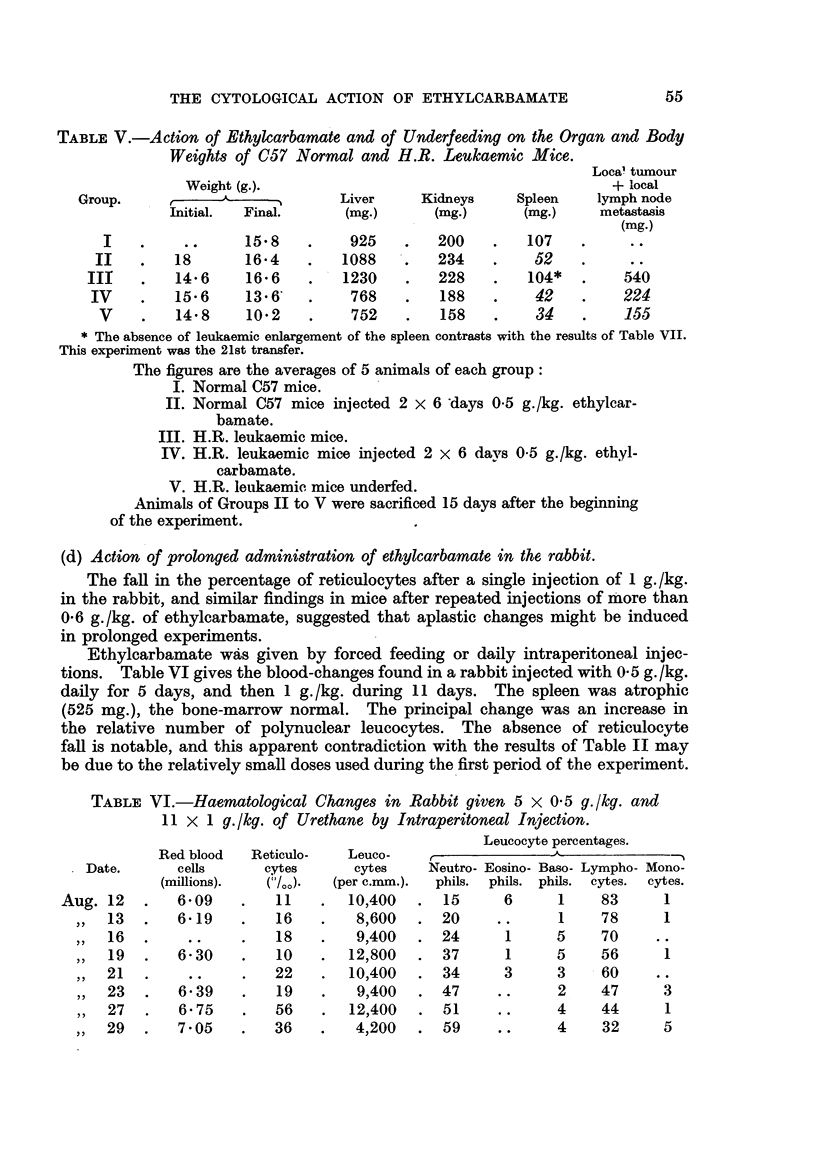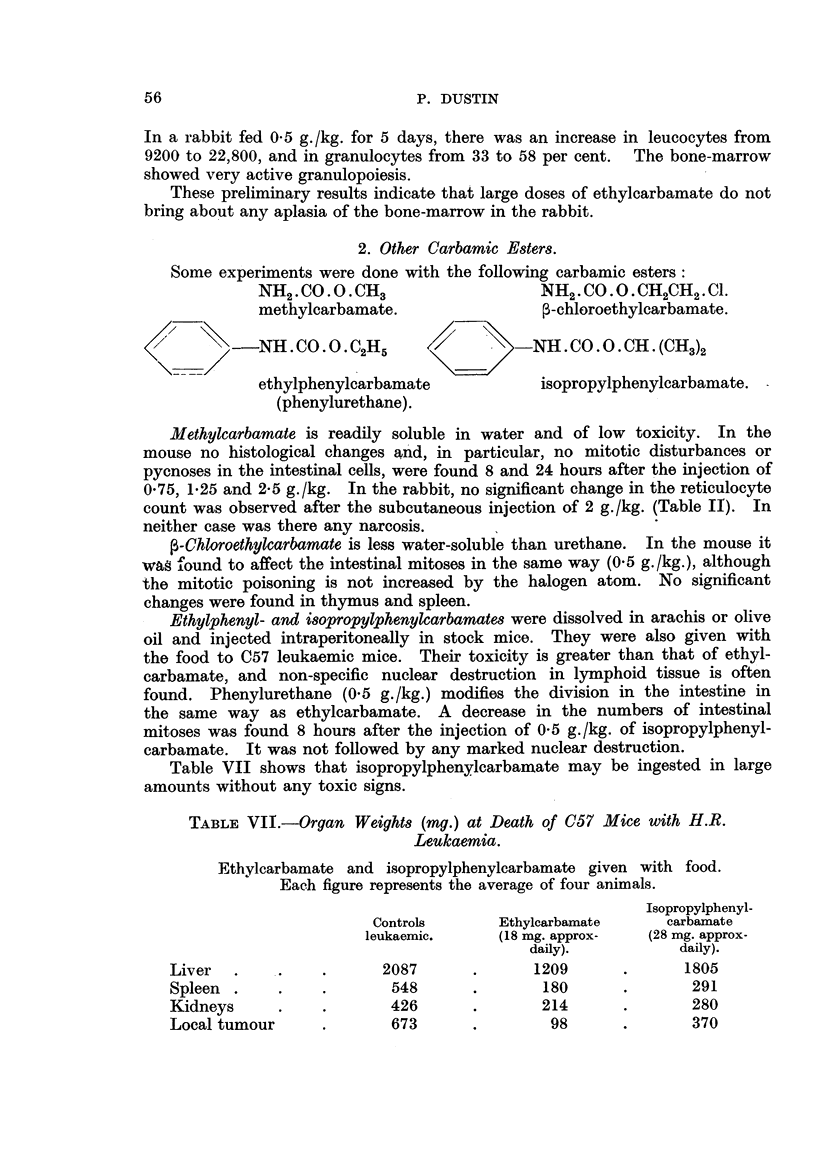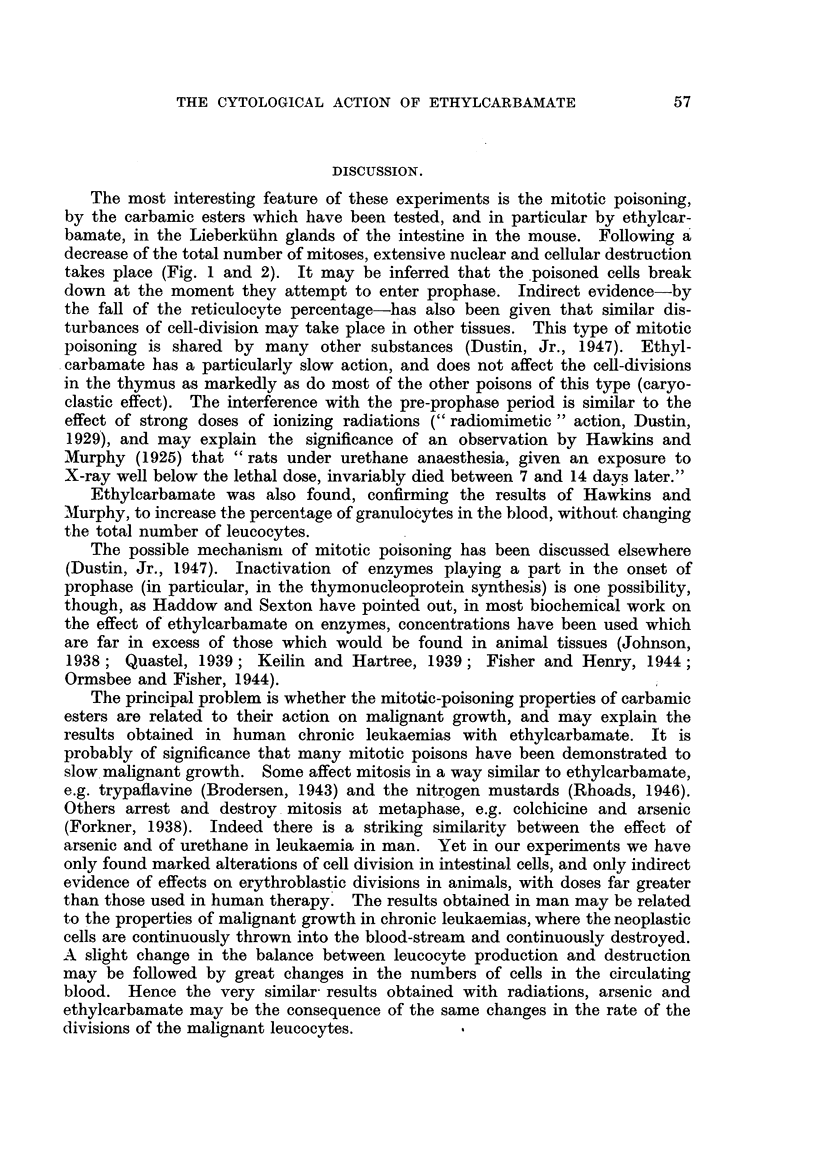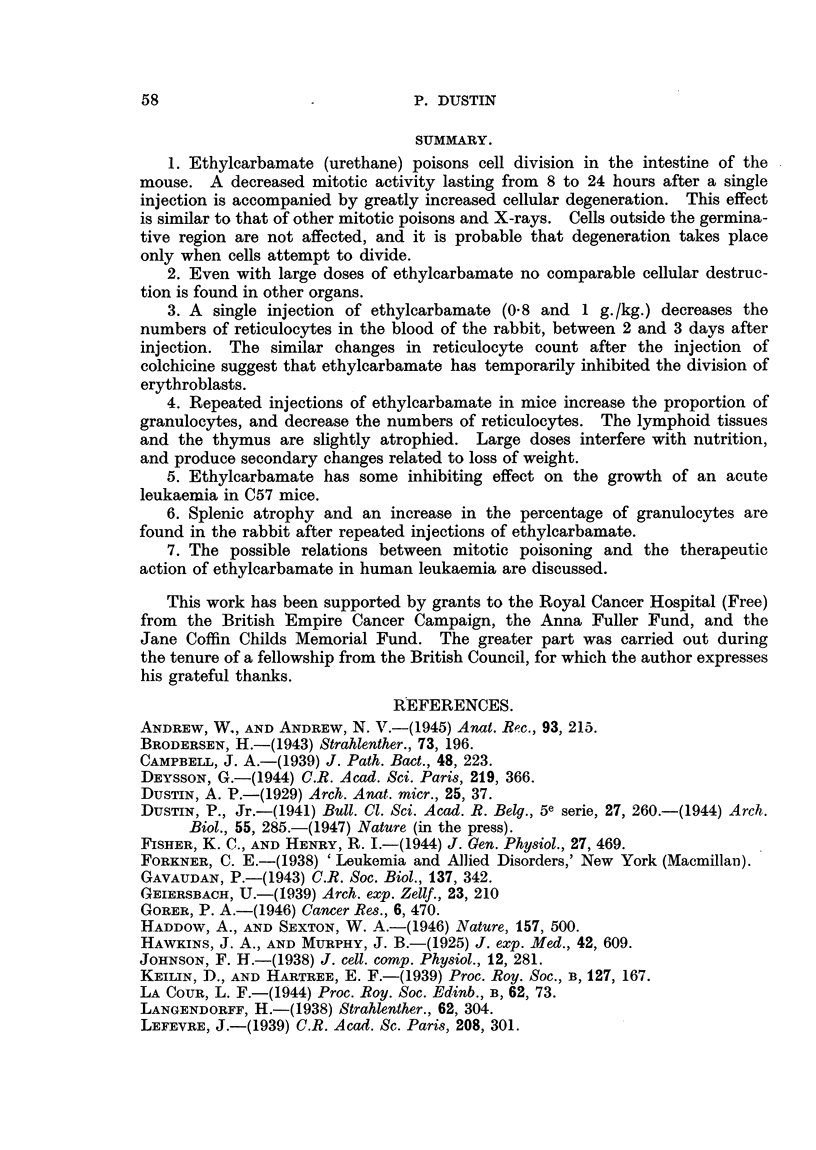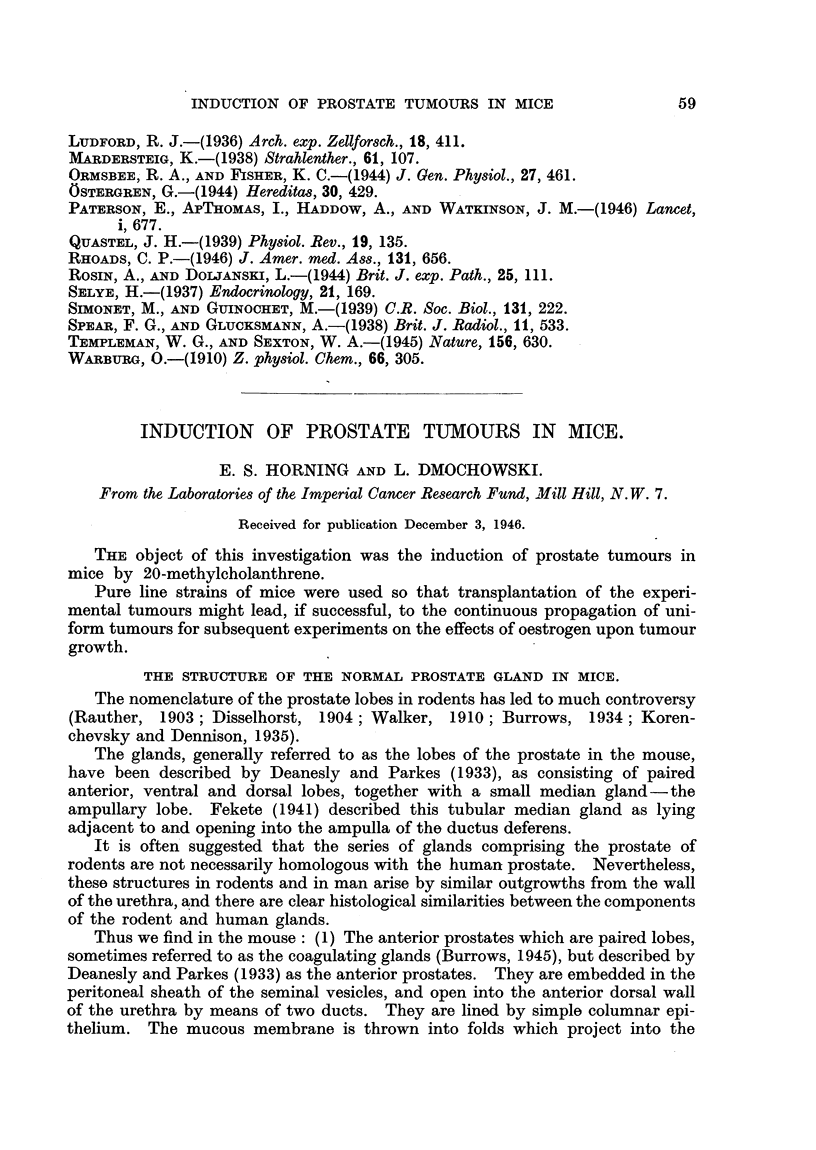# The Cytological Action of Ethylcarbamate (Urethane) and Other Carbamic Esters in Normal and Leukaemic Mice, and in Rabbits

**DOI:** 10.1038/bjc.1947.6

**Published:** 1947-03

**Authors:** P. Dustin

## Abstract

**Images:**


### THE CYTOLOGICAL ACTION OF ETHYLCARBAMATE (URETHANE) AND OTHER CARBAMIC ESTERS IN NORMAL AND LEUKAEMIC MICE, AND IN RABBITS.

P. DUSTIN, JR.*

*From The Chester Beatty Research Institute, The Royal Cancer Hospital (Free), London, S.W. 3.*

Received for publication February 6, 1947.

FOLLOWING the discovery by Haddow and Sexton (1946) of the effects of ethylcarbamate (urethane) on the growth and differentiation of the Walker rat carcinoma, the action of this substance in human leukaemia was described by Paterson, ApThomas, Haddow and Watkinson (1946). Since the early experiments of Warburg in 1910, ethylphenylcarbamate (phenylurethane) had been known to arrest the division of the sea-urchin egg at metaphase. A similar effect in plant cells, closely resembling that of colchicine, was discovered in 1939 by Lefèvre and by Simonet and Guinochet (1939), and was further studied by Gavaudan (1943) and Deysson (1944). Ludford (1936) treated tissue cultures with a 2 per cent solution of ethylcarbamate and noted abnormal metaphases and a considerable amount of nuclear and cellular degeneration. Similar results were obtained by Geiersbach (1939), and Östergren (1944) also claimed that ethylcarbamate arrested mitosis at metaphase in *Allium*. Templeman and Sexton (1945), investigating the growth-inhibiting properties of numerous carbamic esters in plants, found, however, that ethylcarbamate was inactive in this respect, although marked activity was shown by ethyl phenylcarbamate and isopropyl phenylcarbamate. It was this work which led Haddow and Sexton to investigate the action of carbamic esters on tumour growth, and to the discovery that in this case ethylcarbamate itself is effective. These authors suggested that this substance might belong to the group of caryoclastic (nuclear) poisons studied by A. P. Dustin. The purpose of the present paper was to follow this hypothesis. In consequence, the organs in which these caryoclastic effects are known to be most easily observed in mammals (thymus and lymphoid tissue, intestinal mucosa) were specially studied. Experiments were also directed towards investigating the resultant blood changes in normal and leukaemic mice.

### MATERIAL AND METHODS.

Experiments were carried out using stock mice (20–25 g.) and mice of the C57 pure line (*c.* 15 g.). In the latter, an acute leukaemia H.R. described by Gorer (1946) was utilized, in which large stem-cells produced a diffuse infiltration of lymph nodes, spleen, liver and kidneys, leading to death of the mice in from 14 to 22 days after subcutaneous grafting of leukaemic tissue. Enumeration of reticulocytes was made from dry blood smears, following postvital staining with

* Associé du Fonds National Belge de la Recherche Scientifique. Now Lady Tata Memorial Fund Fellow.

FIG. 1.—Intestinal crypts of mouse 10 hours after intraperitoneal injection of 20 mg. (1 g./kg.) of ethylcarbamate. No normal mitoses; extensive nuclear destruction.

FIG. 2.—Intestinal crypts of mouse 24 hours after intraperitoneal injection of 20 mg. (1 g./kg.) of ethylcarbamate. Nuclear destruction and recovering mitosis.

FIG. 3.—Cortical region of the mouse thymus 8 hours after intraperitoneal injection of 50 mg. (2·5 g./kg.) of ethylcarbamate. Normal mitosis; no pycnoses.

brilliant cresyl blue (Dustin, Jr., 1944). Bouin's fluid was used as a routine histological fixative, and cell divisions in the intestine were always studied in the first jejunal loop. For the examination of mitosis in bone marrow, La Cour's (1944) lacmoid-acetic acid staining of squash preparations was found most useful. This method was also applied to other tissues, in particular the intestinal mucosa.

### RESULTS.

### 1. *Ethylcarbamate.*

(a) *Action of a single injection of ethylcarbamate on mitosis in the tissues of adult mice.*

The effects of a single dose of 1 g./kg. of body-weight will first be described. This dose has a narcotic effect lasting more than 3 hours. Freshly-prepared solutions of ethylcarbamate in distilled water were injected intraperitoneally. The most marked changes take place in the germinative zones of the Lieberkühn glands of the small intestine, and these were studied 2, 4, 6, 8, 10, 24 and 48 hours after injection. Three variables must here be considered, namely the mitotic index, the relative percentage of different mitotic phases, and the number of nuclei undergoing degeneration.

(i) One of the best methods for estimating the approximate number of dividing cells in the intestinal mucosa is to count 100 consecutive glandular crypts. This is more reliable than counting the mitoses in a certain number of microscopic fields. In normal mice, in sections 5 to 7$\mu$ thick, 1·2 to 1·5 divisions are found per crypt. After the injection of urethane, this figure does not change during the first 8 hours, but falls to about 0·5 at 10 hours. This decrease in mitotic activity persists 24 hours after the injection. Normal figures are found after 48 hours. Thus ethylcarbamate temporarily lowers the total number of cells undergoing mitosis in the intestine.

(ii) During these experiments there is no evident change in the percentage of different mitotic phases. In one series of mice, prophases varied between 13 and 15 per cent; metaphases, 32 and 52 per cent; ana- and telophases together, 42 and 56 per cent. This indicates that ethylcarbamate does not poison any given phase of cellular division; there is no metaphase arrest of the "colchicine-type." The mitoses which take place are morphologically normal, apart from the possible inclusion of cellular fragments.

(iii) In control animals, occasional pycnotic and shrunken nuclei may be found in the germinative zone of the intestine. Their number increases considerably after a single injection of ethylcarbamate (1 g./kg.):

2 *and* 4 *hours.*—Normal divisions; no degenerating nuclei in abnormal numbers.

6 *hours.*—Slight increase in nuclear destruction.

8 *hours.*—Several pycnotic nuclei in each crypt.

10 *hours.*—Numerous (up to 10 and more) pycnoses per gland (Fig. 1).

24 *hours.*—The nuclear fragments are very numerous. They fuse together and fall into the glandular lumen, being thus eliminated from the epithelium. Fragments are also engulfed in the cytoplasm of normal cells (Fig. 2).

48 *hours*.—The epithelial divisions are again normal, nearly all pycnoses have disappeared, and the only sign of the ethylcarbamate effect is the occasional presence of some voluminous nuclear fragment not yet pushed out into the intestinal cavity.

Nuclear destruction takes place with marked decrease in mitotic index, suggesting that the nuclear fragments originate from cells which normally would have divided. This is substantiated by the cytological analysis of this phenomenon. In normal division, the cells of the intestine undergo the following changes:—*Prophase :* The resting nucleus, lying against the basal membrane, swells progressively and the chromosomes appear close to the nuclear membrane. *Metaphase :* The breakdown of the nuclear membrane is accompanied by marked swelling of the whole cytoplasm, and, as a consequence, the cell migrates towards the glandular lumen. *Ana-* and *Telophase :* The spindle is oriented parallel to the glandular axis. The daughter-nuclei are bean-shaped, and markedly smaller than the resting nuclei* ; they progressively regain the basal zone.

When the nuclei undergo degeneration, the following phases may be observed (Fig. 1): In a cell with a resting nucleus, the cytoplasm undergoes marked swelling. This takes place in a very short time (probably a few minutes), and often brings the cell, as in metaphase, towards the centre of the gland. The nucleus then disintegrates, the thymonucleic acid material forming shell-like structures covering an acidophil sphere, which also contains some ribonucleic acid (by the ribonuclease method) and the remnant of the nucleolus. Further destruction leads to irregular fragments, with a positive or negative Feulgen reaction. These are easily engulfed in the cytoplasm of neighbouring cells, and may sometimes be found in dividing cells.†

Whatever the mitotic poison, these nuclear effects are only to be found in the region where mitosis normally takes place, and the more superficially located resting nuclei are not damaged. This provides evidence that the destruction only affects those cells which would normally have undergone division.

In other tissues no similar changes are to be found. The lymphocytes in the thymus are known to be the most sensitive cells towards mitotic poisons, yet even after injection of 2·5 g./kg. of ethylcarbamate their structure and division appear normal (Fig. 3). In the spleen, neither the divisions of the lymphocytes nor those of the erythroblasts and myelocytes are visibly affected. In the bone-marrow, a study of the mitotic phases was made at repeated intervals after the injection of 1 g./kg. of ethylcarbamate. After 10 hours there were 13 per cent prophases, 63 per cent metaphases and 24 per cent ana- and telophases. After 24 hours the figures were respectively 15, 66 and 19 per cent. The total number of divisions was not estimated and may have varied, as further experiments will show.

The intestinal mitoses appearing to be the most sensitive, an attempt was made to determine the threshold dose of ethylcarbamate. The mitoses and pycnoses were studied 24 hours after the injections, and the results in Table I show that nuclear destruction, already apparently increased by 0·1 g./kg., is considerable after 0·2 g./kg.

* This is perhaps one of the facts which misled Andrew and Andrew (1945) to consider these mitoses as belonging to lymphocytes migrated within epithelial cells.

† This has also been described by Spear and Glücksmann (1938) in the retina of irradiated tadpoles.

No other significant changes were observed in other organs after a single injection of ethylcarbamate.

TABLE I.—*Threshold Action of a Single Intraperitoneal Injection of Ethylcarbamate on the Intestinal Mitoses of the Mouse.*

Each figure represents the total of 100 consecutive glands, at 24 hours following injection.

| | Dose of ethylcarbamate (g./kg.). | | | |
|---|---|---|---|---|
| | 0·20 | 0·15 | 0·10 | 0·05 |
| Mitoses | 147 | 147 | 210 | 181 |
| Pycnotic fragments | 154 | 39 | 23 | 8 |

(b) *Action of a single injection of ethylcarbamate on the number of reticulocytes in rabbit's blood.*

The most sensitive index of the mitotic activity in the red blood cell forming tissues is the reticulocyte percentage in the circulating blood, as pointed out by Langendorff (1938) and Mardersteig (1938) in the study of X-rays on haemopoiesis. The rabbit was chosen as experimental animal for technical reasons, and because marked changes in the reticulocyte percentage had previously been found after the injection of colchicine (Dustin, Jr., 1941).

A single (subcutaneous or intraperitoneal) injection of 0·25 g./kg. of ethylcarbamate did not have any effect, but doses of 0·8 and 1·0 g./kg. produced a definite fall in the number of reticulocytes (Fig. 4 and Table II). In six experiments (five rabbits) this fall was found 48 hours after injection. It was transient,

FIG. 4.

TABLE II.—*Action of Ethyl- and of Methylcarbamate on the Reticulocytes (‰ Red Blood Cells) of Rabbit's Blood.*

| Rabbit No. | Injected (g./kg.). | Days after injection. 0. | 1. | 2. | 3. | 4. | 5. | 6. | 7. | 8. |
|---|---|---|---|---|---|---|---|---|---|---|
| | *Ethylcarbamate.* | | | | | | | | | |
| 3 | 0·5 | 31 | 57 | 30 | 39 | .. | .. | .. | .. | .. |
| 2 | 0·8 | 34 | 30 | *15* | 19 | 20 | 28 | .. | .. | 28 |
| 1 | 1·0 | 16 | 14 | *8* | 9 | 22 | .. | 16 | .. | 12 |
| 2 | 1·0 | 40 | 39 | *24* | 28 | .. | .. | 49 | .. | 52 |
| 5 | 1·0 | 24 | 14 | *16* | *11* | 22 | .. | .. | 20 | .. |
| 6 | 1·0 | 30 | 37 | *12* | *11* | 20 | .. | .. | 21 | .. |
| 7 | 1·0 | 22 | 16 | *13* | 18 | 15 | .. | .. | .. | .. |
| | *Methylcarbamate.* | | | | | | | | | |
| 2 | 2·0 | 17 | 26 | 22 | 24 | .. | .. | .. | .. | .. |
| 3 | 2·0 | 15 | .. | .. | .. | 19 | .. | .. | 18 | .. |

and within 3 days the number of reticulocytes was close to the initial value. No significant change in the numbers of red blood cells, or in the leucocytes, was observed.

These results may be compared with those following the injection of colchicine, when the reticulocyte fall is marked and the minimum is also reached in 48 hours. The erythroblastic mitoses are arrested in metaphase by colchicine during the first hours following its injection, and the delayed decrease of the number of reticulocytes is a consequence of the time needed for the maturation of the erythroblasts. The similarity between the curves for colchicine and for ethylcarbamate suggests that during the first day the narcotic has slowed the production of red blood cells. This is in agreement with the findings for the intestine of the mouse. The fact that neither in rabbits nor in mice can this action of ethylcarbamate be detected by histological methods is only a consequence of the difficulty of estimating the total number of mitoses taking place. The possibility of a mitotic arrest at metaphase is ruled out, but any arrest before prophase would be very difficult to demonstrate by histological methods, especially if there is no extensive cellular breakdown as in the intestine.

(c) *Action of repeated injections in normal and leukaemic C57 mice.*

Apart from their specific effects (for instance mitotic poisoning in the intestine), daily injections of ethylcarbamate may affect the general nutrition of the animals, and cause loss of weight. Underfeeding is known to lead to atrophy of the lymphoid organs (in particular the thymus), and also to slow malignant growth. Hence the great importance of dissociating the direct effects of carbamic esters from non-specific changes related to loss of weight.

(i) *Effect on body-weight.*—Daily injections of 0·25 g./kg. given for a period of two weeks were not found markedly to affect the weight of normal or leukaemic C57 mice. The average loss was about 1 g. for two groups of 5 mice (normal, 17 to 16·3 g.; leukaemic, 19 to 17·9 g.). The same experiment with 0·5 g./kg. daily showed conflicting responses. Some mice lost several grams in a few days, usually dying after losing about one-third of their weight. Others remained in good condition, but lost on the average 2 grams. In a few mice no change of weight was observed. By comparing the histological pictures found in these last two groups it is possible to appreciate the specific effects of ethylcarbamate.

(ii) *Blood-picture.*—The principal changes were an increase in the percentage of granulocytes and a decrease in the numbers of reticulocytes. As could be expected from the results obtained in the rabbit, only doses of 0·5 g./kg. daily changed the reticulocytosis.

Tables III and IV show the influence of repeated injections and ingestion of ethylcarbamate on the reticulocytes of normal and leukaemic C57 mice.

TABLE III.—*Daily Injection of* 0·5 *g./kg. of Ethylcarbamate. Reticulocytes* ‰ *Red Blood Cells* (500 *Cells Counted*).

| Mouse No. | Before 1st injection. | 6 days. | 11 days. | 14 days. | Total loss of weight after 14 days (g.). |
|---|---|---|---|---|---|
| 1 | 28 | 22 | 56 | 96 | 2 |
| 2 | 48 | 11 | 6 | 22 | 4 |
| 3 | 22 | 6 | 28 | 72 | 1 |
| 4 | 64 | 14 | 9 | 38 | 4 |
| 5 | 42 | 16 | 15 | 22 | 1 |

TABLE IV.—*Reticulocytes* (‰ *Red Blood Cells*) *after* 10 *Daily Injections of* 5 *mg. Ethylcarbamate* (c. 0·7 *g./kg.*) *or Feeding with Ethylcarbamate.*

| | Controls normal. | Non-leukaemic fed ethylcarbamate. | Leukaemic fed ethylcarbamate. | Leukaemic injected. | Leukaemic controls. |
|---|---|---|---|---|---|
| | 28 | 23 | 36 | 7 | 114 |
| | 31 | 82 | 13 | 6 | 82 |
| | 54 | 18 | 14 | 22 | 84 |
| | 72 | 10 | 17 | 6 | 62 |
| | 34 | 16 | 7 | 10 | 100 |
| Average | 44 | 30 | 17½ | 10 | 88½ |

This was most conspicuous in leukaemic animals, where the reticulocytosis associated with the neoplasia was no longer visible (Table IV). Table III shows that the reticulocyte fall may be transient, and followed by a rise even when the daily administration of ethylcarbamate is pursued. It did not appear to be related to loss of body weight, as is the following effect. With daily doses exceeding 0·5 g./kg., extremely low reticulocyte numbers (sometimes less than 1‰) were found, both in normal and leukaemic mice. This was not an indication of irreversible damage to red blood cell production, and after cessation of the injections a high reticulocytosis was found. The blood of normal C57 mice contains about 30 per cent of granulocytes, and a similar proportion is found during the leucocytosis associated with the H.R. leukaemia. In 1925 Hawkins and Murphy found that urethane anaesthesia was followed by a relative increase in the percentage of granulocytes in rabbits. Both in normal and leukaemic mice the continued administration of ethylcarbamate has the same result. Though individual differences may be great, in all animals treated with at least 0·5 g./kg./day, the percentage of granulocytes was increased after 15 injections to an average figure of 60 per cent both in normal and leukaemic mice. The change is quite conspicuous after 6 daily injections. No apparent relation was found between the percentage of granulocytes and the total number of leucocytes, which, particularly in leukaemic animals, varied considerably (between normal figures averaging 5000, and 120,000/c.mm.).

(iii) *Organ weights and histology.*—Table V shows clearly that both in normal and leukaemic animals ethylcarbamate produces a marked atrophy of the spleen, which is associated with thymic atrophy. There is some evidence that the growth of the H.R. leukaemic cells is also disturbed, as evidenced by the smaller size of the tumour at the site of grafting and the less conspicuous enlargement of liver, spleen and kidneys by neoplastic infiltration. This effect seems partly non-specific, and can be produced by underfeeding alone.

The following histological changes are to be found in mice given repeated injections of ethylcarbamate (0·5 g./kg.) for 10 to 15 days without loss of weight:

*Thymus :* the cortical zone is thinner than normal, and its limits are less precise. Normal mitoses are found in the cortex, and there is no evidence of increased pycnotic destruction of thymocytes.

*Spleen :* the atrophy of the organ affects all haemopoietic tissues. Germ-centres are rarely present in the lymph follicles. Formation of granulocytes, megakaryocytes and red blood cells is still taking place.

*Lymph-nodes :* slight atrophy.

*Bone-marrow* (femur): very active granulopoiesis. No evidence of aplasia or atrophy.

*Liver, Kidneys, Adrenals :* no histological change.*

*Intestine :* much pycnotic debris in all Lieberkühn crypts, side by side with normal mitoses. No evident atrophy of the mucosa, nor ulceration.

*Leukaemic infiltrations :* no evidence of increased cell-destruction nor mitotic abnormalities.

In animals losing weight during the experiment, splenic and lymphoid atrophy is more pronounced. The formation of granulocytes and red blood cells in the spleen is markedly reduced. The bone-marrow remains very cellular, with a high proportion of immature granulocytes. There is no evidence of aplasia. In the thymus the atrophy of the cortical region is complete, the epithelial cells only remaining. This picture of " inverted thymus " is found after the pycnotic destruction of thymus cells by mitotic poisons (Dustin, 1929) or non-specific stimuli (Selye, 1937), and in inanition. In this case it is probable that the ethylcarbamate injections and the loss of body weight have additive effects.

No marked changes are found in other organs.

* Rosin and Doljanski (1944) have described inclusions in liver cells of rats treated with urethane, which they believe are red blood cells engulfed in cytoplasm and even in the nucleus. From the evidence given, the origin of these inclusions is doubtful. Similar bodies were described in the liver cells of the mouse by Campbell (1939) and observed occasionally in our material.

TABLE V.—*Action of Ethylcarbamate and of Underfeeding on the Organ and Body Weights of C57 Normal and H.R. Leukaemic Mice.*

| Group. | Weight (g.). Initial. | Final. | Liver (mg.) | Kidneys (mg.) | Spleen (mg.) | Loca' tumour + local lymph node metastasis (mg.) |
|---|---|---|---|---|---|---|
| I | .. | 15·8 | 925 | 200 | 107 | .. |
| II | 18 | 16·4 | 1088 | 234 | *52* | .. |
| III | 14·6 | 16·6 | 1230 | 228 | 104* | 540 |
| IV | 15·6 | 13·6 | 768 | 188 | *42* | *224* |
| V | 14·8 | 10·2 | 752 | 158 | *34* | *155* |

* The absence of leukaemic enlargement of the spleen contrasts with the results of Table VII. This experiment was the 21st transfer.

The figures are the averages of 5 animals of each group :

I. Normal C57 mice.

II. Normal C57 mice injected 2 × 6 days 0·5 g./kg. ethylcarbamate.

III. H.R. leukaemic mice.

IV. H.R. leukaemic mice injected 2 × 6 days 0·5 g./kg. ethylcarbamate.

V. H.R. leukaemic mice underfed.

Animals of Groups II to V were sacrificed 15 days after the beginning of the experiment.

(d) *Action of prolonged administration of ethylcarbamate in the rabbit.*

The fall in the percentage of reticulocytes after a single injection of 1 g./kg. in the rabbit, and similar findings in mice after repeated injections of more than 0·6 g./kg. of ethylcarbamate, suggested that aplastic changes might be induced in prolonged experiments.

Ethylcarbamate was given by forced feeding or daily intraperitoneal injections. Table VI gives the blood-changes found in a rabbit injected with 0·5 g./kg. daily for 5 days, and then 1 g./kg. during 11 days. The spleen was atrophic (525 mg.), the bone-marrow normal. The principal change was an increase in the relative number of polynuclear leucocytes. The absence of reticulocyte fall is notable, and this apparent contradiction with the results of Table II may be due to the relatively small doses used during the first period of the experiment.

TABLE VI.—*Haematological Changes in Rabbit given 5 × 0·5 g./kg. and 11 × 1 g./kg. of Urethane by Intraperitoneal Injection.*

| Date. | Red blood cells (millions). | Reticulocytes (°/₀₀). | Leucocytes (per c.mm.). | Leucocyte percentages. Neutrophils. | Eosinophils. | Basophils. | Lymphocytes. | Monocytes. |
|---|---|---|---|---|---|---|---|---|
| Aug. 12 | 6·09 | 11 | 10,400 | 15 | 6 | 1 | 83 | 1 |
| ,, 13 | 6·19 | 16 | 8,600 | 20 | .. | 1 | 78 | 1 |
| ,, 16 | .. | 18 | 9,400 | 24 | 1 | 5 | 70 | .. |
| ,, 19 | 6·30 | 10 | 12,800 | 37 | 1 | 5 | 56 | 1 |
| ,, 21 | .. | 22 | 10,400 | 34 | 3 | 3 | 60 | .. |
| ,, 23 | 6·39 | 19 | 9,400 | 47 | .. | 2 | 47 | 3 |
| ,, 27 | 6·75 | 56 | 12,400 | 51 | .. | 4 | 44 | 1 |
| ,, 29 | 7·05 | 36 | 4,200 | 59 | .. | 4 | 32 | 5 |

In a rabbit fed 0·5 g./kg. for 5 days, there was an increase in leucocytes from 9200 to 22,800, and in granulocytes from 33 to 58 per cent. The bone-marrow showed very active granulopoiesis.

These preliminary results indicate that large doses of ethylcarbamate do not bring about any aplasia of the bone-marrow in the rabbit.

### 2. *Other Carbamic Esters.*

Some experiments were done with the following carbamic esters :

$NH_2.CO.O.CH_3$ methylcarbamate.

$NH_2.CO.O.CH_2CH_2.Cl.$ β-chloroethylcarbamate.

$—NH.CO.O.C_2H_5$

ethylphenylcarbamate (phenylurethane).

$—NH.CO.O.CH.(CH_3)_2$

isopropylphenylcarbamate.

*Methylcarbamate* is readily soluble in water and of low toxicity. In the mouse no histological changes and, in particular, no mitotic disturbances or pycnoses in the intestinal cells, were found 8 and 24 hours after the injection of 0·75, 1·25 and 2·5 g./kg. In the rabbit, no significant change in the reticulocyte count was observed after the subcutaneous injection of 2 g./kg. (Table II). In neither case was there any narcosis.

*β-Chloroethylcarbamate* is less water-soluble than urethane. In the mouse it was found to affect the intestinal mitoses in the same way (0·5 g./kg.), although the mitotic poisoning is not increased by the halogen atom. No significant changes were found in thymus and spleen.

*Ethylphenyl- and isopropylphenylcarbamates* were dissolved in arachis or olive oil and injected intraperitoneally in stock mice. They were also given with the food to C57 leukaemic mice. Their toxicity is greater than that of ethylcarbamate, and non-specific nuclear destruction in lymphoid tissue is often found. Phenylurethane (0·5 g./kg.) modifies the division in the intestine in the same way as ethylcarbamate. A decrease in the numbers of intestinal mitoses was found 8 hours after the injection of 0·5 g./kg. of isopropylphenylcarbamate. It was not followed by any marked nuclear destruction.

Table VII shows that isopropylphenylcarbamate may be ingested in large amounts without any toxic signs.

TABLE VII.—*Organ Weights (mg.) at Death of C57 Mice with H.R. Leukaemia.*

Ethylcarbamate and isopropylphenylcarbamate given with food. Each figure represents the average of four animals.

| | Controls leukaemic. | Ethylcarbamate (18 mg. approx. daily). | Isopropylphenylcarbamate (28 mg. approx. daily). |
|---|---|---|---|
| Liver | 2087 | 1209 | 1805 |
| Spleen | 548 | 180 | 291 |
| Kidneys | 426 | 214 | 280 |
| Local tumour | 673 | 98 | 370 |

### DISCUSSION.

The most interesting feature of these experiments is the mitotic poisoning, by the carbamic esters which have been tested, and in particular by ethylcarbamate, in the Lieberkühn glands of the intestine in the mouse. Following a decrease of the total number of mitoses, extensive nuclear and cellular destruction takes place (Fig. 1 and 2). It may be inferred that the poisoned cells break down at the moment they attempt to enter prophase. Indirect evidence—by the fall of the reticulocyte percentage—has also been given that similar disturbances of cell-division may take place in other tissues. This type of mitotic poisoning is shared by many other substances (Dustin, Jr., 1947). Ethylcarbamate has a particularly slow action, and does not affect the cell-divisions in the thymus as markedly as do most of the other poisons of this type (caryoclastic effect). The interference with the pre-prophase period is similar to the effect of strong doses of ionizing radiations (" radiomimetic " action, Dustin, 1929), and may explain the significance of an observation by Hawkins and Murphy (1925) that " rats under urethane anaesthesia, given an exposure to X-ray well below the lethal dose, invariably died between 7 and 14 days later."

Ethylcarbamate was also found, confirming the results of Hawkins and Murphy, to increase the percentage of granulocytes in the blood, without changing the total number of leucocytes.

The possible mechanism of mitotic poisoning has been discussed elsewhere (Dustin, Jr., 1947). Inactivation of enzymes playing a part in the onset of prophase (in particular, in the thymonucleoprotein synthesis) is one possibility, though, as Haddow and Sexton have pointed out, in most biochemical work on the effect of ethylcarbamate on enzymes, concentrations have been used which are far in excess of those which would be found in animal tissues (Johnson, 1938 ; Quastel, 1939 ; Keilin and Hartree, 1939 ; Fisher and Henry, 1944 ; Ormsbee and Fisher, 1944).

The principal problem is whether the mitotic-poisoning properties of carbamic esters are related to their action on malignant growth, and may explain the results obtained in human chronic leukaemias with ethylcarbamate. It is probably of significance that many mitotic poisons have been demonstrated to slow malignant growth. Some affect mitosis in a way similar to ethylcarbamate, e.g. trypaflavine (Brodersen, 1943) and the nitrogen mustards (Rhoads, 1946). Others arrest and destroy mitosis at metaphase, e.g. colchicine and arsenic (Forkner, 1938). Indeed there is a striking similarity between the effect of arsenic and of urethane in leukaemia in man. Yet in our experiments we have only found marked alterations of cell division in intestinal cells, and only indirect evidence of effects on erythroblastic divisions in animals, with doses far greater than those used in human therapy. The results obtained in man may be related to the properties of malignant growth in chronic leukaemias, where the neoplastic cells are continuously thrown into the blood-stream and continuously destroyed. A slight change in the balance between leucocyte production and destruction may be followed by great changes in the numbers of cells in the circulating blood. Hence the very similar results obtained with radiations, arsenic and ethylcarbamate may be the consequence of the same changes in the rate of the divisions of the malignant leucocytes.

SUMMARY.

1. Ethylcarbamate (urethane) poisons cell division in the intestine of the mouse. A decreased mitotic activity lasting from 8 to 24 hours after a single injection is accompanied by greatly increased cellular degeneration. This effect is similar to that of other mitotic poisons and X-rays. Cells outside the germinative region are not affected, and it is probable that degeneration takes place only when cells attempt to divide.

2. Even with large doses of ethylcarbamate no comparable cellular destruction is found in other organs.

3. A single injection of ethylcarbamate (0·8 and 1 g./kg.) decreases the numbers of reticulocytes in the blood of the rabbit, between 2 and 3 days after injection. The similar changes in reticulocyte count after the injection of colchicine suggest that ethylcarbamate has temporarily inhibited the division of erythroblasts.

4. Repeated injections of ethylcarbamate in mice increase the proportion of granulocytes, and decrease the numbers of reticulocytes. The lymphoid tissues and the thymus are slightly atrophied. Large doses interfere with nutrition, and produce secondary changes related to loss of weight.

5. Ethylcarbamate has some inhibiting effect on the growth of an acute leukaemia in C57 mice.

6. Splenic atrophy and an increase in the percentage of granulocytes are found in the rabbit after repeated injections of ethylcarbamate.

7. The possible relations between mitotic poisoning and the therapeutic action of ethylcarbamate in human leukaemia are discussed.

This work has been supported by grants to the Royal Cancer Hospital (Free) from the British Empire Cancer Campaign, the Anna Fuller Fund, and the Jane Coffin Childs Memorial Fund. The greater part was carried out during the tenure of a fellowship from the British Council, for which the author expresses his grateful thanks.

REFERENCES.

Andrew, W., and Andrew, N. V.—(1945) *Anat. Rec.*, **93**, 215.
Brodersen, H.—(1943) *Strahlenther.*, **73**, 196.
Campbell, J. A.—(1939) *J. Path. Bact.*, **48**, 223.
Deysson, G.—(1944) *C.R. Acad. Sci. Paris*, **219**, 366.
Dustin, A. P.—(1929) *Arch. Anat. micr.*, **25**, 37.
Dustin, P., Jr.—(1941) *Bull. Cl. Sci. Acad. R. Belg.*, 5e serie, **27**, 260.—(1944) *Arch. Biol.*, **55**, 285.—(1947) *Nature* (in the press).
Fisher, K. C., and Henry, R. I.—(1944) *J. Gen. Physiol.*, **27**, 469.
Forkner, C. E.—(1938) 'Leukemia and Allied Disorders,' New York (Macmillan).
Gavaudan, P.—(1943) *C.R. Soc. Biol.*, **137**, 342.
Geiersbach, U.—(1939) *Arch. exp. Zellf.*, **23**, 210
Gorer, P. A.—(1946) *Cancer Res.*, **6**, 470.
Haddow, A., and Sexton, W. A.—(1946) *Nature*, **157**, 500.
Hawkins, J. A., and Murphy, J. B.—(1925) *J. exp. Med.*, **42**, 609.
Johnson, F. H.—(1938) *J. cell. comp. Physiol.*, **12**, 281.
Keilin, D., and Hartree, E. F.—(1939) *Proc. Roy. Soc.*, B, **127**, 167.
La Cour, L. F.—(1944) *Proc. Roy. Soc. Edinb.*, B, **62**, 73.
Langendorff, H.—(1938) *Strahlenther.*, **62**, 304.
Lefevre, J.—(1939) *C.R. Acad. Sc. Paris*, **208**, 301.

LUDFORD, R. J.—(1936) *Arch. exp. Zellforsch.*, **18**, 411.
MARDERSTEIG, K.—(1938) *Strahlenther.*, **61**, 107.
ORMSBEE, R. A., AND FISHER, K. C.—(1944) *J. Gen. Physiol.*, **27**, 461.
ÖSTERGREN, G.—(1944) *Hereditas*, **30**, 429.
PATERSON, E., APTHOMAS, I., HADDOW, A., AND WATKINSON, J. M.—(1946) *Lancet*, i, 677.
QUASTEL, J. H.—(1939) *Physiol. Rev.*, **19**, 135.
RHOADS, C. P.—(1946) *J. Amer. med. Ass.*, **131**, 656.
ROSIN, A., AND DOLJANSKI, L.—(1944) *Brit. J. exp. Path.*, **25**, 111.
SELYE, H.—(1937) *Endocrinology*, **21**, 169.
SIMONET, M., AND GUINOCHET, M.—(1939) *C.R. Soc. Biol.*, **131**, 222.
SPEAR, F. G., AND GLUCKSMANN, A.—(1938) *Brit. J. Radiol.*, **11**, 533.
TEMPLEMAN, W. G., AND SEXTON, W. A.—(1945) *Nature*, **156**, 630.
WARBURG, O.—(1910) *Z. physiol. Chem.*, **66**, 305.